# P048. Thunderclap headache as presentation of ischemic posterior circulation stroke

**DOI:** 10.1186/1129-2377-16-S1-A173

**Published:** 2015-09-28

**Authors:** Martina Bruno, Matteo Atzori, Anna Maria Basile, Giorgio Zanchin, Ferdinando Maggioni

**Affiliations:** Headache Centre of Veneto Region, Department of Neurosciences, Padua University, Padua, Italy; Stroke Unit and Neurosonology Laboratory, St. Anthony Hospital, Padua, Italy

## Introduction

We describe the case of a man who presented with an episode fulfilling the ICHD-III criteria for thunderclap headache (TH), whose diagnostic work-up led to an unexpected diagnosis.

## Case report

**Figure 1:**
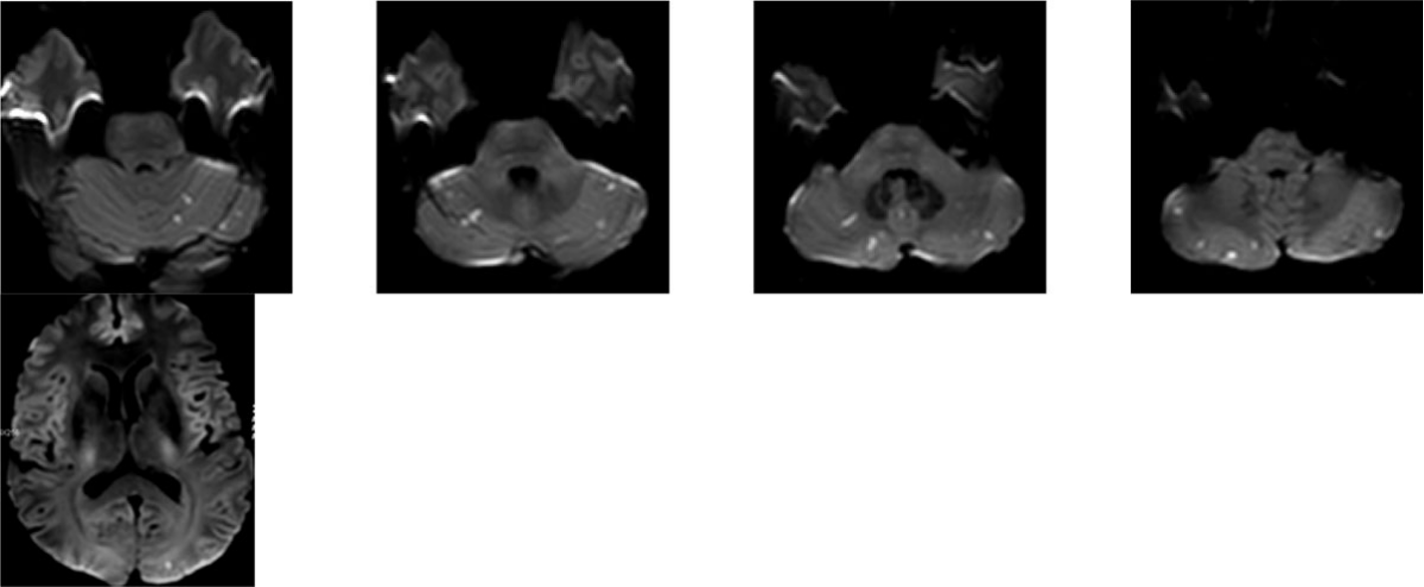

A 49-year-old man presented after the onset of a sudden throbbing laterocervical right pain, rapidly spreading bilaterally to his head, reaching the maximum of its intensity in 1 minute. Headache was associated with nausea, dizziness, blurred vision and bilateral tinnitus. Patient's past medical history was significant for hypertension, hypercholesterolemia and obstructive sleep apnoea syndrome. He did not report any recent head traumas, physical efforts or consumption of vasoactive drugs. He had recently undergone chiropractor procedures. At arrival to our institution, nausea and blurred vision had resolved, a mild bilateral tinnitus, as well as mild frontal and occipital headache persisted. Neurological examination was unremarkable, particularly he had no neck stiffness, neither photophobia. Brain CT and neurovascular ultrasound examination were normal. An MR of the brain with MR Angiography (MRA) disclosed any dissection but revealed multiple small recent ischemic lesions in the cerebellar hemispheres and in the left occipital lobe (Figure 1).

He underwent a CT angiography that confirmed MRA findings. Laboratory analysis, including hypercoagulable tests, was normal. He was treated immediately with antiplatelet drugs and he has been asymptomatic ever since. Echocardiography and transcranial color Doppler with bubble test were normal. He underwent a second MRA that did not show any intramural hematoma suggestive for artery dissection. He was discharged after one week and further tests were scheduled, including transesophageal echocardiography and Holter-EKG.

## Discussion

There have been an expanding number of conditions associated to TH, including cerebral venous sinus thrombosis, cervical artery dissection, and reversible vasoconstriction syndrome [1]. Approximately 25% of patients with stroke develop an associated headache, but is often overshadowed by the abrupt neurological deficits. In these cases the pathophysiology for headache is multifactorial and involves the direct activation of nociceptive sensory afferents innervating the intracranial vasculature, the release of vasoactive neuropeptides from sensory afferents and of inflammatory cytokines from damaged tissue. This is one of the rare reports of a TH as the presenting and primary clinical feature in an ischemic cerebellar stroke, in the absence of neurological findings [2]. The addition of ischemic stroke expands the differential diagnosis of TH and reinforces the need for MR imaging, even when the initial neurological examination, brain CT, are unrevealing.

Written informed consent to publish was obtained from the patient(s).

## References

[1] Ducros A, Bousser MG (2013). Thunderclap headache. BMJ.

[2] Schwedt TJ, Dodick DW (2006). Thunderclap stroke: embolic cerebellar infarcts presenting as thunderclap headache. Headache.

